# Quantitative evaluation of posture control in rats with inferior olive lesions

**DOI:** 10.1038/s41598-021-99785-w

**Published:** 2021-10-13

**Authors:** Tetsuro Funato, Yota Sato, Yamato Sato, Soichiro Fujiki, Shinya Aoi, Kazuo Tsuchiya, Dai Yanagihara

**Affiliations:** 1 Department of Mechanical Engineering and Intelligent Systems, The University of Electro-Communications, 1-5-1 Chofugaoka, Chofu, Tokyo 182-8585 Japan; 2 Department of Life Sciences, The University of Tokyo, 3-8-1 Komaba, Meguro-ku, Tokyo, 153-8902 Japan; 3 Faculty of Creative Engineering, Chiba Institute of Technology, 2-1-1 Shibazono, Narashino, Chiba 275-0023 Japan; 4 Department of Physiology, Dokkyo Medical University, 880 Kitakobayashi, Mibu, Shimotsugagun, Tochigi 321-0293 Japan; 5 Department of Aeronautics and Astronautics, Kyoto University, Kyoto daigaku-Katsura, Nishikyo-ku, Kyoto, 615-8540 Japan

**Keywords:** Dynamical systems, Motor control, Cerebellum

## Abstract

Impairment of inferior olivary neurons (IONs) affects whole-body movements and results in abnormal gait and posture. Because IONs are activated by unpredicted motion rather than regular body movements, the postural dysfunction caused by ION lesions is expected to involve factors other than simple loss of feedback control. In this study, we measured the postural movements of rats with pharmacological ION lesions (IO rats) trained to stand on their hindlimbs. The coordination of body segments as well as the distribution and frequency characteristics of center of mass (COM) motion were analyzed. We determined that the lesion altered the peak properties of the power spectrum density of the COM, whereas changes in coordination and COM distribution were minor. To investigate how the observed properties reflected changes in the control system, we constructed a mathematical model of the standing rats and quantitatively identified the control system. We found an increase in linear proportional control and a decrease in differential and nonlinear control in IO rats compared with intact rats. The dystonia-like changes in body stiffness explain the nature of the linear proportional and differential control, and a disorder in the internal model is one possible cause of the decrease in nonlinear control.

## Introduction

Damage to inferior olivary neurons (IONs) affects whole-body movements and results in abnormal gait and posture. Animals with pharmacological^1,2^ or genetic^3^ lesions to the IONs show exaggerated flexion of the limbs, an abnormal shift of the center of mass (COM) motion, and limb ataxia^1,2^, as well as dystonia-like movements with stiff limbs and tremor^3^. Motor function assessment with the rotating rod test identified reduced retention time on the rod and changes in postural maintenance behavior in rats with ION lesions (IO rats)^4^. We wondered how ION lesions impair postural maintenance.

Loss of signals derived from IONs increases the spontaneous firing activities of simple spikes in Purkinje neurons, resulting in abnormal oscillations that periodically repeat with bursts and brief offsets^5^. In mice with dystonic behavior due to ION impairment, inhibition of the highly irregular firing activities of the cerebellar nuclei eliminates the tremor and improves movement^3^. This suggests that the dystonic behavior caused by the abnormal firing activities of Purkinje neurons is critical to ION lesion-related postural dysfunction. On another front, whether the movement abnormality caused by the dystonic behavior is the main factor in reducing stability or whether there are other factors has yet to be resolved.

Signals from IONs contribute multiple functions to postural control, and olivo-cerebellar system lesions possibly cause multiple impairments related to postural dysfunction. The first possible impairment is related to the stiff limbs and tremor. The aforementioned mice with genetically impaired IONs show dystonia-like symptoms^3^. Abnormalities in limb stiffness and muscle tone increase the difficulty of movement control and can lead to postural movement instability. The second possible impairment concerns timing and coordination. IONs contribute to the regulation of movement timing and thereby coordinate multiple movements, such as breathing, swallowing, and licking during water drinking^6,7^. Cats with ION lesions exhibit limb ataxia^1,2^, and the loss of coordination may affect their postural stability. The third possible impairment is related to the internal model and the consequent motor program. ION-derived signals are responsible for sending error signals to the cerebellum^8,9^. The error signals are used to construct an internal model in the cerebellum^10,11^. The internal model has been suggested to contribute to postural control^12^, and olivo-cerebellar system lesions may affect control based on the internal model.

To reveal the contribution of olivo-cerebellar system lesions to the destabilized posture, it would be useful to quantitatively evaluate the control system through the measurement of the movement of animals with olivo-cerebellar system lesions and a comparison with a mathematical model of the control system. In previous work, we constructed an experimental system for measuring the movement of rats with bipedal standing^13^. Because bipedal standing is unstable, rats must move for stabilization. Here, the rats’ motion will include the characteristics of posture control. By investigating the standing motion of IO rats and identifying the control system from their motion, we can address the stabilized and destabilized mechanisms of posture control.

In this study, we adopted the following approach: (1) intact and IO rats (created using 3-acetylpyridine and nicotinamide injection) were maintained in the bipedal upright posture and their motions were measured; (2) the coordination among joints was analyzed from the measured motions; (3) the distribution and power spectra of the COM position were derived to explore the changes in behavior required to maintain the COM; and (4) the control system was identified from the COM behavior and the changes in the control system caused by a lesion in the olivo-cerebellar system were quantitatively evaluated.

## Results

### Lesion condition of inferior olive neurons

**Figure 1:**
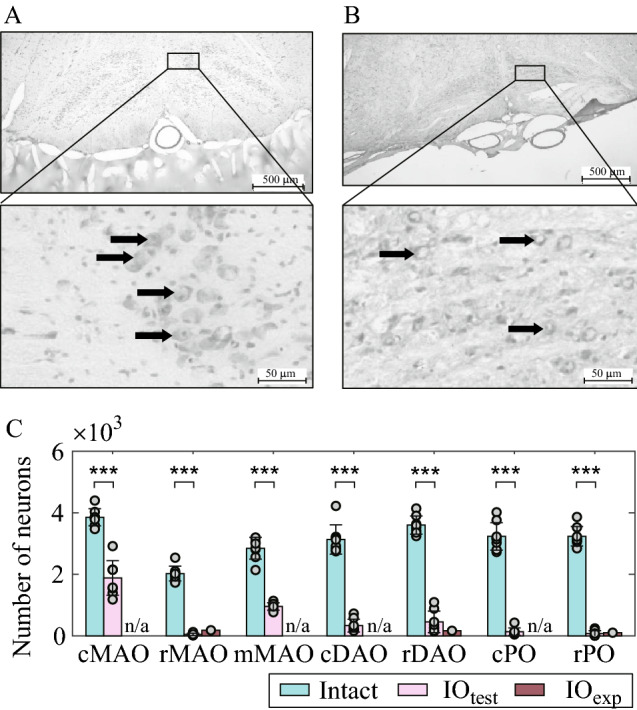
Lesion condition of inferior olive neurons. (**A**) Cerebellar section of intact rats. (**B**) Cerebellar section of IO rats. Arrows in the figure represent inferior olive neurons. (**C**) Number of surviving neurons in intact (n = 8) and IO (IO_test_: n = 8; and IO_exp_: n = 1) rats. Bars, error bars, and points are the mean values, standard deviation, and individual data, respectively. IO_test_ represents the IO rats that were used only for this test of the lesion condition and were not used in the standing experiment. IO_exp_ represents the rat used for the standing experiment. Surviving neurons were counted in seven subdivisions of the inferior olivary nucleus: cMAO, mMAO, and rMAO, caudal, middle, and rostral divisions of the medial accessory olive; cDAO and rDAO, caudal and rostral divisions of the dorsal accessory olive; cPO and rPO, caudal and rostral divisions of the principal olive. ***P < 0.001. Neurons in the rMAO, rDAO, and rPO were counted for IO_exp_, and n/a represents data not available.

To investigate the postural control dysfunction induced by a lesion in the olivo-cerebellar system, we created rats with an ION lesion (IO rats) through intraperitoneal administration of 3-acetylpyridine (3-AP) and nicotinamide. We subsequently evaluated the pharmacological destruction of the IONs by counting the number of surviving neurons in brain slices. Figure 1 shows the imaging data of the brain sections of an intact rat (Fig. 1A) and an IO rat (Fig. 1B). The circular objects indicated by arrows in the figure represent the IONs, with the dots representing their nuclei. Figure 1A displays a large number of intact neurons with their nuclei. On the other hand, Fig. 1B shows a general decrease in neurons and multiple neurons without nuclei. These figures confirm that the drug administration led to an ION lesion.

Figure 1C show the number of surviving neurons in brain sections. This number does not include neurons without nuclei. We counted the neuron numbers of eight intact rats (Intact), eight IO rats (IOtest), and another IO rat that was subjected to the posture experiment (IOexp). Surviving neurons were counted for seven subdivisions of the inferior olive^14^: cMAO, mMAO, and rMAO, caudal, middle, and rostral divisions of the medial accessory olive; cDAO and rDAO, caudal and rostral divisions of the dorsal accessory olive; cPO and rPO, caudal and rostral divisions of the principal olive. Two-way ANOVA of rat type (Intact/IOtest) and IO subdivision showed significant effects of both rat type (P < 0.001, f = 1044.5, df = 1) and subdivision (P < 0.001, f = 27.1, df = 6). Moreover, a *t*-test comparison of intact and IOtest in each subdivision obtained P < 0.001 for all subdivisions (t = 8.9, 23.0, 14.3, 15.2, 19.4, 18.9, and 27.1 for cMAO, rMAO, mMAO, cDAO, rDAO, cPO, and rPO, respectively; df = 14 for all subdivisions), indicating the effect of the drug administration on the neurons in all of the seven IO subdivisions. Figure 1C also shows that the individual differences are not large. Moreover, we did not find any specific differences in the behavior of each IOtest rat. In the IOexp, the surviving neurons were counted for three rostral subdivisions that were drastically affected by 3-AP: rMAO, rDAO, and rPO. Figure 1C shows that the numbers of surviving neurons in the IO rats used for the posture experiment (IOexp) were also similar to those in the other IO rats (IOtest). These results validated the induction of the olivo-cerebellar lesion in the IO rats used for our experiment.

### Standing behavior in IO rats

We measured the standing behavior of intact and IO rats (created using 3-AP and nicotinamide injection) encouraged to stand upright on only their two hindlimbs. Their steady standing movement was measured for more than 60 s in 11 trials of six intact rats and in 10 trials of four IO rats. Intact rats maintained a relatively steady standing posture, whereas IO rats oscillated back and forth during standing and abruptly lost the standing posture (see Supplementary Video S1).

Reflective markers were attached near the joints of the rats, and a motion capture system measured the movements of the joints. We analyzed the data obtained to investigate the effects of the lesion on the coordination of body segments and the movement of the COM (see Supplementary Text, Supplementary Fig. S1, and Supplementary Table S1 for a comparison between COM and center of pressure). Here, to avoid the multiple comparisons problem, we selected the data of one trial for each rat and used these values for the statistical analysis and figures (see “Statistical analysis” in “Methods” for an explanation of trial selection).

### Segmental coordination, COM distribution, and power spectrum density

**Figure 2:**
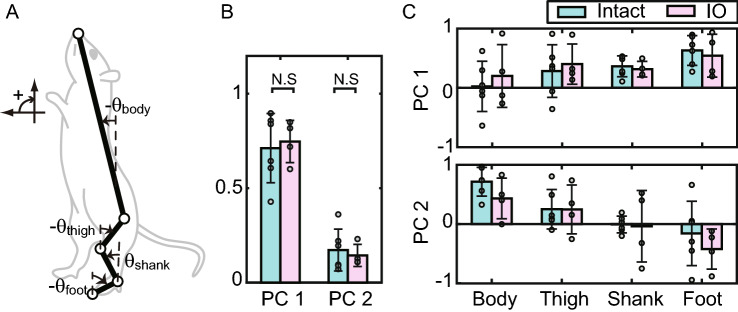
Intersegmental coordination of intact and IO rats. (**A**) Elevation angles of body segments. (**B**) Contribution ratio. (**C**) Intersegmental coordination. PC 1 and PC 2 represent the first and second principal components determined by singular value decomposition, respectively. Each bar represents the average and standard deviation of intact (n = 6) and IO (n = 4) rats. Each point represents individual data. N.S., not significant.

We calculated the elevation angles of the four body segments֫—body, thigh, shank, and foot (Fig. 2A)—from the measured motions and applied singular value decomposition to investigate the coordination among body segments. Singular value decomposition of the matrix of segmental angles derives the principal components (PCs) of the matrix. We named the PC with the largest contribution PC 1 and that with the second largest contribution PC 2, and so on. The PC of the body segments represents a motion element with a fixed ratio among body segments, and this motion element represents the coordination among segments. Previous studies of standing in both humans^15^ and intact rats^13^ showed that mainly just two PCs contribute to bipedal standing. The study that assessed the coordination of intact rats^13^ also indicated that PC 1 was strongly correlated with COM movements, whereas PC 2 was correlated mainly with trunk movements. Figure 2 shows the contribution ratio (Fig. 2B) and the components of the segmental coordination (Fig. 2C) of intact and IO rats. The cumulative contribution ratios of the two types of coordination (PC 1 and PC 2) were 0.88 (± 0.07) in the intact rats and 0.95 (± 0.01) in the IO rats, indicating that the standing motions comprised two patterns, similar to the previous results^13,15^. There was no significant difference in the contribution ratio (Fig. 2B) between intact and IO rats (PC 1: P = 0.74, t =  − 0.34, df = 8; PC 2: P = 0.65, t = 0.47, df = 8; *t*-test). Figure 2C shows the components of the segmental coordination, with similar patterns for intact and IO rats. To assess the significance of the differences in intersegmental coordination (Fig. 2C) between intact and IO rats, we performed a two-way ANOVA of segment and rat type (intact and IO). No significant difference was found between the components of the intact and IO rats in both PC 1 and PC 2 (PC 1: P = 0.72, f = 0.13, df = 1; PC 2: P = 0.24, f = 1.45, df = 1). These results indicate that an ION lesion does not significantly alter the coordination for standing movements, at least at the level of segmental motion.

**Figure 3:**
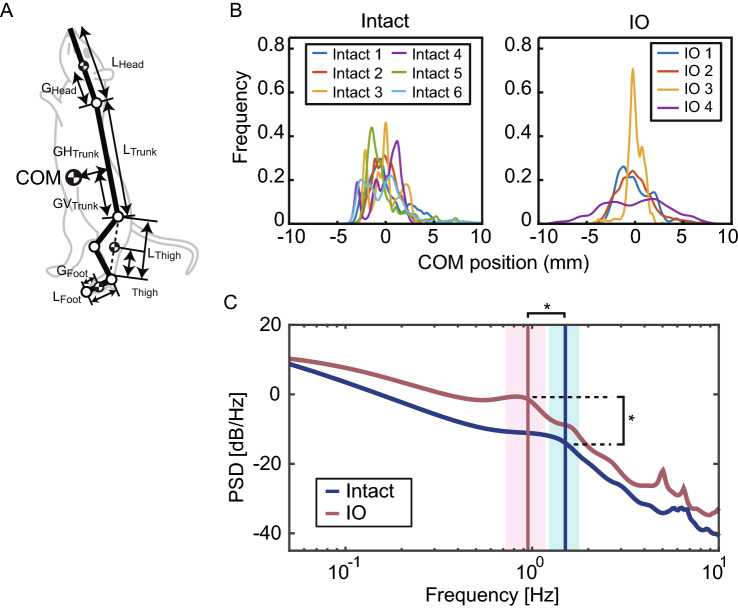
Center of mass (COM) characteristics in intact and IO rats. (**A**) Center of mass. *L* of each body segment represents the length of the segments, and *G* of each segment represents the length from the end point to the COM of the segment. (**B**) Probability density function (PDF) of the COM. The PDF was calculated using kernel distribution with the mean value of the COM set to 0. Each curve in B with a different color represents a different trial (Intact, n = 6; IO, n = 4). (**C**) Power spectrum density (PSD) of the COM. Each curve represents the mean value of the PSD in intact (n = 6) and IO (n = 4) rats. The vertical lines and colored areas in (**C**) represent the mean and standard deviation of the peak frequencies, respectively. *, P < 0.05.

Next, we calculated the COM motion on the sagittal plane and investigated the probability density function (PDF) of the horizontal COM motion. In our previous work^13^, we measured the ratio between the COM of each body segment and marker position anatomically (*G/L* for each body segment in Fig. 3A). We used these ratios and the measured marker motions of the standing rats to calculate the COM. Figure 3B shows the PDF of the intact and IO rats, which reveals the distribution of the COM motion on the sagittal plane. The figure indicates similar PDFs for intact and IO rats. To evaluate the similarity in the PDF between intact and IO rats, we calculated the standard deviation (SD) and kurtosis of the COM. The SDs of the COM position were 1.85 (± 0.50) (mm) for intact rats and 2.00 (± 1.00) (mm) for IO rats, with no significant difference (P = 0.76, t =  − 0.32, df = 8 in the *t*-test, P = 1.00 in the Wilcoxon rank-sum test). The kurtosis values of the COM position were 3.85 (± 1.83) for intact rats and 4.48 (± 1.96) for IO rats, with no significant difference (P = 0.62, t =  − 0.52, df = 8 in the *t*-test, P = 0.61 in the Wilcoxon rank-sum test). These results also indicate that the PDF of the COM was not significantly different between intact and IO rats.

Finally, the power spectrum density (PSD) of the horizontal COM in the sagittal plane was calculated (Fig. 3C). The average PSDs of intact and IO rats are shown in Fig. 3C. The vertical lines and regions in Fig. 3C are the average values and SDs of the peak frequencies, respectively. As shown in the figure, the lowest peak frequencies were at about 1 Hz in both intact and IO rats. The peak frequencies were 1.50 (± 0.28) Hz for intact rats and 0.94 (± 0.23) Hz for IO rats. To investigate the effect of the lesion on the PSDs of the COM, we compared the peak frequencies and the powers of the peak frequencies between intact and IO rats. The results showed that the peak frequency was significantly lower (P = 0.01, t = 3.32, df = 8, *t*-test) and the power significantly higher (P = 0.03, t =  − 2.66, df = 8, *t*-test) in IO rats than in intact rats. These results indicate that an ION lesion affects the PSD of the COM, in particular, for motion with peak frequencies at about 1 Hz.

### Model of the posture control system in rats

**Figure 4:**
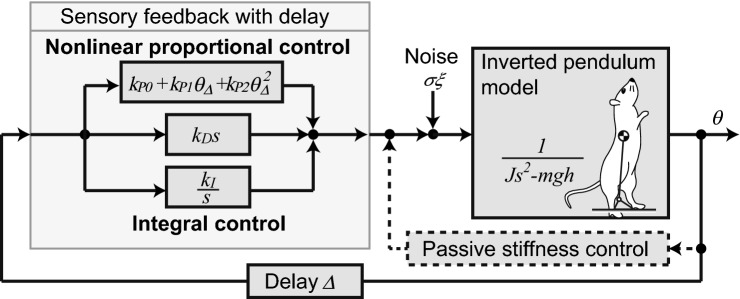
Block diagram of the mathematical model of standing control in rats. The body of the rat is modeled by a single-link inverted pendulum and controlled by delayed sensory feedback. The control system comprises a nonlinear proportional–integral–derivative (PID) control.

Our experiments showed that an ION lesion had a significant effect on the PSD of the COM in standing control, with no significant effect on the segmental coordination or the distribution of the COM. To determine what changes in the postural control system reflected the changes in the PSD, we evaluated the control system using a mathematical model. Figure 4 shows a mathematical model of the body and the control system of the standing rats. In the model, the body of the rat is represented as an inverted pendulum with one link from the ankle to the COM, and the posture control system is represented by a third-order nonlinear feedback control. Various physiological properties, such as the contribution of visual, vestibular, and somatosensory information to posture control^16,17^ (including in patients with Parkinson's disease^18^), effect of body stiffness^19,20^, and the mechanism generating large body sway with physiological noise^21,22^, have been evaluated using models based on proportional–integral–derivative (PID) control (see also Methods). By using such a PID-based model to evaluate the control system of rats, we can discuss the results from the perspective of the previous evaluations of physiological properties. However, it should be noted that our rat experiment evaluates the effect of the lesion on ION function, and it is not obvious whether the PID-based model explains the dysfunction with ION lesions, such as that related to the internal model. In particular, prediction is an important control feature^23^ that can be built with the internal model and that can be used for posture control. Thus, we first examined how the nature of the predictive control relates to the third-order nonlinear PID control (Fig. 4).

When predictive control is assumed in the control system, the posture control is a hybrid control of the predictive control and the stabilization function by muscle stiffness (reflex-mediated stiffness control^19^). Here, the stiffness control works as a linear PD control based on its spring and damping characteristics. Because ION lesions produce dystonia-like changes in body stiffness^3^, stiffness control is largely affected by such lesions. Meanwhile, the effect of prediction on quiet standing is not obvious. Thus, the variation in the linear part of the hybrid control will be accounted for by the change in the stiffness. In other words, the influence of predictive control appears mainly in the nonlinear control portion. Any nonlinear control term, including predictive control, can be decomposed into polynomial nonlinear terms by Taylor expansion (as long as it takes the form of sensory feedback). The third-order nonlinear control (Fig. 4) corresponds to the approximation by terms up to the third order in the Taylor expansion^24^. Thus, our control model (Fig. 4) considers a low-dimensional approximate model of hybrid control including predictive control. Therefore, this model can potentially be used to evaluate the properties of the control system, including predictive control, in an approximate manner.

To investigate the behavior of the model (Fig. 4), we entered the physical parameters of the rats, such as rat height and weight, into the model and investigated the effect of the control system on the PSD. An analytical solution was obtained with the approximation of no noise and the integral control gain *k*_*I*_ ≅ 0. Then, a periodic solution with the following angular frequency *ω* and amplitude *r* was obtained (see Supplementary Methods for details).

1 $$\begin{array}{*{20}c} {{\upomega }^{2} \simeq \frac{{k_{D} - mgh{\Delta }}}{{{\Delta }\left( {J - {\Delta }k_{D} } \right)}}} \\ \end{array}$$

2 $$\begin{array}{*{20}c} {{\text{r}}^{2} \simeq \frac{{k_{D} - k_{P0} {\Delta }}}{{3k_{P2} {\Delta }}}} \\ \end{array}$$

where *m*, *h*, *J*, and *g* are the mass of the rat, length from ankle to COM, moment of inertia, and gravitational acceleration, respectively. *k*_*P0*_, *k*_*P2*_, and *k*_*D*_ are the linear proportional control gain, nonlinear control gain, and differential control gain, respectively. *Δ* is the sensory delay, and we set *Δ* = 0.04 s based on previous research^25^.

**Figure 5:**
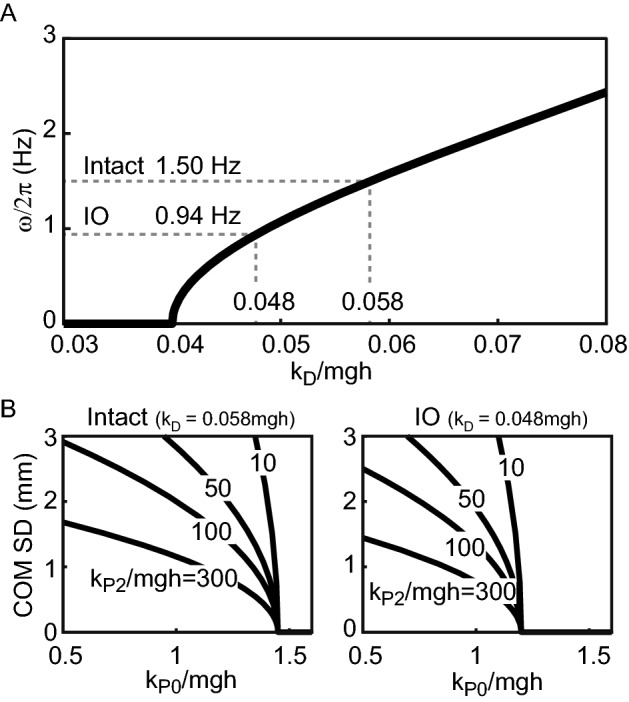
Properties of the control parameters derived from mathematical analysis of the model. (**A**) Relationship between the differential control gain and the frequency of the body sway. The dotted line in the figure represents the average peak frequencies in the power spectrum density of the experiment (intact, 1.50 Hz; IO, 0.94 Hz) and the corresponding differential control gain *k*_*D*_. (**B**) Relationship between the linear proportional control gain *k*_*P0*_, the nonlinear control gain *k*_*P2*_, and the amplitude of the body sway (standard deviation of the center of mass [COM SD]). Here, the values of the differential control gain *k*_*D*_ for intact and IO rats were taken from the values in (**A**).

Substituting the mean body weight and body length of the rats—*m* = 220.3 g and *h* = 73.2 mm—into Eq. (1) produced an oscillation of about 1 Hz at *k*_*D*_ ≅ 0.05*mgh* (Fig. 5A). In particular, substituting the average peak frequencies of the experimentally obtained rat power spectra—1.50 Hz (intact rats) and 0.94 Hz (IO rats)—resulted in differential control gains of *k*_*D*_ = 0.058*mgh* (intact rats) and *k*_*D*_ = 0.048*mgh* (IO rats), respectively. Thus, the decrease in the peak frequency due to the ION lesion reflected the decrease in the differential control gain *k*_*D*_.

Next, we examined the relationship between the amplitude of the PSD and the control parameters. The differential control gain obtained from Fig. 5A (*k*_*D*_ = 0.058*mgh* and *k*_*D*_ = 0.048*mgh*) and the rats’ body weight and body length were substituted into Eq. (2) to give the relationship between the proportional control gains *k*_*P0*_ and *k*_*P2*_ and the COM amplitude *r*. The SD of the horizontal COM movement (COM SD) was then calculated from the amplitude of the COM angle *r* (see “Methods”). Figure 5B shows the relationship between the SD of the COM and the linear proportional control gain *k*_*P0*_ at nonlinear control gains *k*_*P2*_ = 300*mgh*, 100*mgh*, 50*mgh*, and 10*mgh*. When *k*_*P0*_ falls below the threshold of approximately *mgh*, COM oscillation results (Funato et al*.*^24^ shows this mechanism), and the amplitude of the oscillation increases with a decreasing *k*_*P0*_. A larger nonlinear control gain *k*_*P2*_ suppresses the amplitude of the oscillation when *k*_*P0*_ is smaller than the threshold. Therefore, the increase in the amplitude seen in the IO rats reflects a decrease in the linear or nonlinear proportional control gain.

### Identification of the posture control system

Using a mathematical model with the above characteristics, we quantitatively evaluated the control system based on the experimental data. The PDF and PSD of the COM were calculated by a numerical simulation of the model including noise and delay. Then, we searched for the control parameters of the model whose PDF and PSD of the COM matched those of the experiments. The parameters considered were the linear proportional control gain *k*_*P0*_, nonlinear control gain *k*_*P2*_, differential control gain *k*_*D*_, integral control gain *k*_*I*_, and magnitude of the noise σ.

We identified the parameters of each trial using the Generic Algorithm. The identification was repeated ten times with different random seeds, and the optimal parameter set with the smallest value of the evaluation function was selected as the control parameters. The convergence of the parameter search was verified by comparing the variation in obtained parameters between ten identification trials and the variation in parameters between each rat. The SDs of the ten identification trials were smaller than those of the rats for all parameters (see Supplementary Fig. S2). We used a two-way ANOVA with identification trial and rats. Significant differences were not found among the identified trials for any of the trials (see Fig. S1 for the ANOVA result), whereas significant differences were found among the rats (P < 0.001), except for *k*_*I*_ in the IO rats (P = 0.98). These results indicate that the identification results were sufficiently convergent and that the values of the identified parameters were likely to reflect the characteristics of the experimental data.

**Figure 6:**
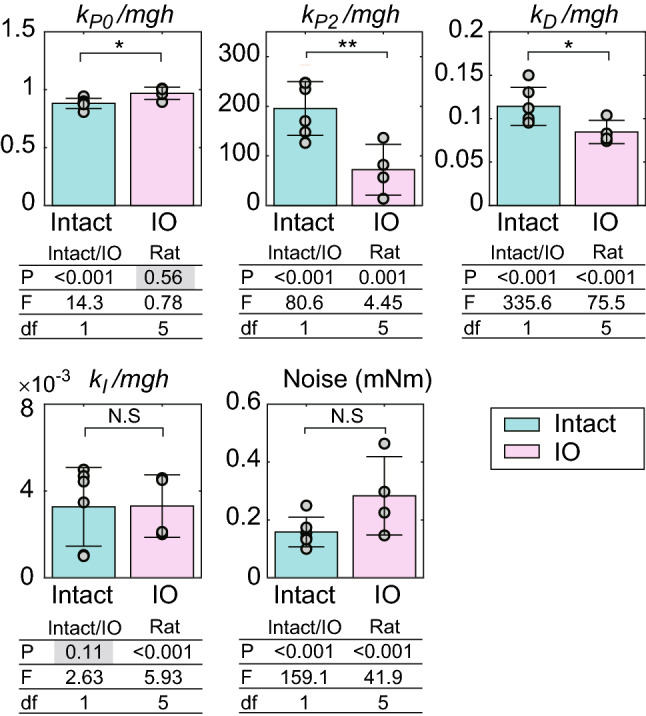
Identified control parameters. The figures show the result of the linear control gain *k*_*P0*_, nonlinear control gain *k*_*P2*_, differential control gain *k*_*D*_, integral control gain *k*_*I*_, and magnitude of noise. Parameter values are normalized by *mgh* (*m* = mass, *g* = acceleration due to gravity, and *h* = body height). Each bar shows the average and standard deviation of the results in intact (n = 6) and IO (n = 4) rats. Each point represents individual data. *, P < 0.05; **, P < 0.01; N.S., not significant with a *t*-test. The tables below the bar charts show the P values (P), F values (F), and degrees of freedom (df) of two-way ANOVA with rat type (Intact/IO) and individual rats (Rat). P values exceeding 0.05 are written with a gray background.

Figure 6 shows the results of the identified control parameters. Integral control gain *k*_*I*_ (P = 0.98, t =  − 0.03, df = 8, *t*-test) and noise σ (P = 0.07, t =  − 2.10, df = 8, *t*-test) were not significantly different between intact and IO rats, but other parameters were significantly different. For example, nonlinear control gain *k*_*P2*_ (P = 0.007, t = 3.61, df = 8, *t*-test) and differential control gain *k*_*D*_ (P = 0.044, t = 2.39, df = 8, *t*-test) were decreased in IO rats compared with intact rats. In contrast, linear proportional control gain *k*_*P0*_ (P = 0.023, t =  − 2.82, df = 8, *t*-test) was increased in IO rats. Two-way ANOVA with rat type and individual rats supported the significant difference in the parameters between the rat types, including the lack of a significant difference in integral control gain (Fig. 6). These results indicate that the functions underlying linear proportional control, differential control, and nonlinear control were altered by an ION lesion and that these changes are responsible for the reduced stability.

## Discussion

The present study analyzed the posture of bipedally standing rats and investigated changes in postural control systems associated with a lesion in the olivo-cerebellar system. Both intact and IO rats (created using 3-AP and nicotinamide injection) were able to stand for more than 60 s on only their hindlimbs. We successfully measured the standing movement of the rats, which includes the movement for posture stabilization. IO rats showed a wobble during standing and suddenly lost their balance and fell down (see Supplementary Video S1). We analyzed the intersegmental coordination and the PDF and PSD of the COM. We failed to find a significant effect of the lesion on the intersegmental coordination and the PDF of the COM but did find a significant effect on the PSD of the COM. In particular, the peak frequency and the amplitude of the peak frequency were significantly different between intact and IO rats. To reveal how the changes in the PSD reflect the effects of the postural control system, a posture control model of the rat was constructed and a quantitative evaluation of the control parameters was performed based on the experimental data. The results revealed that IO rats showed lower nonlinear and differential control gains and higher linear proportional control gain and noise compared with intact rats. We will now discuss the properties of lesions in the olivo-cerebellar system that are related to the change in the identified parameters and address the mechanism of postural dysfunction due to a lesion in the olivo-cerebellar system.

A limitation of the present approach for investigating the function of ION lesions is that IO rats were created by the injection of healthy rats with 3-AP and nicotinamide. The 3-AP intoxication protocol, which was established in 1970s^1^, is still in use today^26–28^. Injection of 3-AP with nicotinamide results in highly selective lesioning of the ION^1,27^. However, indirect effects of 3-AP have also been reported^29^. The potentially affected areas include hypoglossal neurons^1^, the nucleus ambiguous, the dorsal motor nucleus, the nucleus dorsalis raphe, the interpeduncular nucleus, the substantia nigra, and the hippocampal formation^29^. Most areas do not have direct effects on posture control during standing, but the substantia nigra and nigrostriatal pathway play a substantial role in posture control. Degeneration of these areas causes a deficiency in dopamine and leads to Parkinson’s disease^30^. In patients with Parkinson’s disease, the PSD lower than 0.7 Hz was reported to decrease as a characteristic standing behavior^31^. In contrast, the PSD of our rats showed high power in the lower frequency range (Fig. 3C). This supports our belief that the effect of 3-AP on the substantia nigra and nigrostriatal pathway was minor. Research also indicates that ION lesions affect the activity of the cerebellar system^32^. This implies that the change in the behavior of IO rats is partially caused by the inactivity of the system, not only the ION lesion itself. Electrophysiological experiments may provide insight into such an effect of inactivity of the cerebellar system. One approach to more directly assess the effect of the ION lesion is to use an optogenetic method^3^. The optogenetic method is well-established for mice but not for rats. Because mice are too active to maintain a standing posture, we used rats and 3-AP to create and evaluate ION lesions in the present work. Use of the optogenetic method to address the posture control of standing rats is a promising avenue for future research.

IONs send a climbing fiber to Purkinje neurons, and the excitatory inputs from the climbing fiber induce complex spike activities in Purkinje neurons. IONs fire with slow frequencies^33^, and the firing rate of complex spikes in Purkinje neurons increases when motion is not predicted^34,35^. These characteristics of ION activity suggest that the postural dysfunction caused by ION lesions involves factors other than simple loss of feedback control. Indeed, linear proportional control gain was not decreased in IO rats in the present study and other factors must thus be considered to explain the postural instability.

One of the functions of the olivo-cerebellar system that may affect posture control is their role in movement coordination. IONs have been reported to comprise a network structure in the cerebellar system using subthreshold oscillations and to have the ability to synchronize and coordinate with the output of cerebellar activities^36^. Olivo-cerebellar system lesions cause impaired coordination^1,37^ and we thus hypothesized that the impaired coordination could affect standing control. We accordingly examined the intersegmental coordination during standing. However, we were unable to identify a significant difference in coordination between intact and IO rats (Fig. 2), suggesting at least that impaired coordination is not the critical reason for the postural dysfunction in the standing rats.

Lesions in the olivo-cerebellar system affect properties related to body stiffness. For example, cats with rostral medial accessory olive inactivation show limping and waddling, in addition to a wobbly figure-eight trunk, during gait^38,39^. The loss of signals derived from IONs causes abnormal oscillations that periodically repeat with bursts and brief offsets in Purkinje neurons^5^. In mice with dystonic behavior, inhibition of the highly irregular firing activities of the cerebellar nuclei eliminates the tremor and improves movement^3^. This suggests that loss of IONs causes a stiff body via abnormal oscillations in the cerebellar nuclei. From the perspective of the control system, a stiff body represents an increase in the modulus of elasticity; during postural control during standing, the linear proportional control gain of the feedback control system corresponds to this modulus of elasticity. Oscillation of the body is suppressed by the viscosity of the body and, conversely, oscillation is more likely to occur when the viscosity decreases. The viscosity of the body corresponds to the differential control gain of the feedback control system. Therefore, the increase in linear proportional control and the decrease in differential control seen in the results from the IO rats are considered to reflect dystonia-like behavior.

The next aspect to be discussed is how these increases in linear proportional control and decreases in differential control affect stability. An increase in linear proportional control generally helps to suppress body sway. Indeed, cyclic COM motion is generated during standing when linear proportional gain becomes lower than *k*_*P0*_ ≅ *mgh* and the COM amplitude increases with a decreasing linear proportional gain if other parameters are fixed (Fig. 5B). On the other hand, increased linear proportional control or body stiffness simultaneously makes the body more sensitive to sensory delays and sensory errors. For patients with spinocerebellar ataxia, reduced knee flexion and hypermetria in the pelvis and trunk were reported as factors of destabilization during standing^40^, with increased linear proportional control possibly involved in the destabilization. Differential (velocity) control was indicated to contribute to stability by compensating for the effect of a sensory delay^41^. This is because the velocity control responds faster than positional control. Muscles are reported to be activated prior to the motion during standing^42^, and this prior activity is suggested to reflect the presence of differential control^43^. In other words, a decrease in differential control is likely to cause instability due to a sensory delay. In summary, we suggest one of the factors underlying the instability in the IO rats to be the dystonia-like changes in body motion, which decrease robustness due to a stiff body and weak differential control.

The control function that showed a significant decrease in gain in IO rats was nonlinear control. The nonlinear control used in this study was a proportional control with third-order nonlinearity. This nonlinear control generates a strong control force for recovery when the posture is largely tilted. Thus, the identified decrease in the nonlinear control suggests a dysfunction of this recovery function, leading to destabilization of the posture. The third-order nonlinear control applied in this study is an approximate model of nonlinear control used to ensure analysis and identification, and there are several possibilities for the actual nonlinear control structure. One possible type of nonlinear control for standing involves an internal model. IONs send error signals concerning motion to the cerebellum^8,9^, where an internal model for the motion is created^10,11^. The involvement of internal models in posture control is supported by a predictive behavior in posture control, called anticipatory postural adjustment (APA)^23^. APA is an involuntary movement that precedes voluntary movement to support stability, and the APA is reported to use the predicted posture at the time of movement execution or at the end of the movement^44^. Previous reports showed that humans and animals use a body scheme—an internal representation of the body—to predict movement^45,46^. Other work demonstrated that the internal forward model^47^ necessary for predicting movement relies on the activity of Purkinje cells in the cerebellum^48,49^. One possible control scheme involving prediction is model predictive control (MPC)^50^. In MPC, future movement with a given control is predicted for a certain range, and the control command is determined to optimize the predicted movement. Let us consider the control command determined by the MPC and consider whether the control command with the MPC is related to the control command with our nonlinear control. The MPC can send an optimized command for stabilization with minimum torque: the control torque is small when the body is quasivertical (with low possible instability) and large when the sway angle is large (with high possible instability). This implies that control based on predictions using the internal model may behave similarly to the nonlinear control evaluated in this study. Therefore, a disorder in the internal model associated with an ION lesion is potentially reflected as a decrease in the identified nonlinear control.

Above all, the IO rats exhibited reduced controllability of the body due to dystonia-like changes in body stiffness and a lower correction capability of large body sway due to a decrease in nonlinear control, which would be caused by a disorder in the internal model. These changes in the posture control system are considered to cause the instability with sudden loss of balance exhibited by IO rats.

## Methods

### Animals

Six healthy male Wistar rats, aged 4–6 months, weighing 200.8 (± 18.1) g, and with a body height during bipedal standing of 158.1 (± 17.8) mm, were used for the experiments with intact rats. Four male Wistar rats, aged 4–7 months, weighed 229.2 (± 52.1) g, and with a body height during bipedal standing of 167.0 (± 12.1) mm, were used for the experiments with IO rats. One rat (labeled as Intact1 and IO1 in the Results) was used both as an intact and an IO rat, and other rats were used as either intact or IO rats. In total, nine rats were used for the standing experiments. Supplementary Table S2 shows the body mass and height of each rat. To create ION lesions, 3-acetylpyridine (3-AP) was intraperitoneally administered at 75 mg/kg body weight^1^, which was followed 3.5 h later by nicotinamide at 300 mg/kg body weight^14,51^.

Before the measurement, the intact rats underwent bipedal standing training until an observer visually confirmed that they could stand bipedally. The same training was also performed for all IO rats before lesion induction (so that the experience of standing was roughly equal for all IO rats). About 1 month after drug administration, the rats restarted the bipedal standing training for 1 to 2 weeks. After we confirmed that the IO rats could stand bipedally for a certain period of time, we started the measurement.

To confirm the drug-induced ION lesion, we sliced and stained the brains of the rats after all of the experiments (see also the Supplementary Methods). We then visually counted the numbers of surviving IONs in the brain sections and compared the numbers of surviving neurons between intact and IO rats. The validations were performed on 17 rats: one IO rat used in the standing experiments, eight intact rats, and eight IO rats that were not used in the standing experiments (i.e., 16 additional rats were used for confirmation of the ION lesion).

### Motion experiments and analysis

To investigate the effect of an ION lesion on stabilization-related movement, rats stood with only their hindlimbs for more than 60 s, and their movement was measured. The experimental environment was constructed in our previous study^13^. A motion capture system was used to measure the rats’ movement. The measurement frequency was 500 Hz.

From the motions measured by the motion capture system, we investigated the intersegmental coordination, PDF, and PSD of the COM. The coordination relationships of body segments (intersegmental coordination) were extracted from the matrix with the time series of four segmental angles using singular value decomposition (SVD)^13^. The four body segments were the trunk, thigh, shank, and foot. The ratio of segmental angles for composing the intersegmental coordination and the contribution rate of each intersegmental coordination were compared between intact and IO rats.

COM during standing was calculated from the measured joint motion and the COM characteristics of the rat determined in our previous study^13^. The PDF of the COM position in the sagittal plane was derived to compare the distribution of the COM between intact and IO rats. The frequency characteristics of body sway were evaluated using the PSD of the COM. The PSD was calculated using the maximum entropy method (MEM)^52^. We identified the peak frequencies of the PSD using the “findpeaks” function in MATLAB.

### Mathematical model of posture control and identification

Mathematical models for human standing have been explored in various studies, with most using an inverted pendulum with one or two links for the body model and feedback control for the control model^16,53,54^. Analysis of standing motion in intact rats^13^ (and IO rats in Fig. 2) showed that the motion in the sagittal plane mainly comprised two types of movements and, similar to humans, the movement of the COM accounted for most of the movements. Therefore, we modeled the body motion of the standing rat using an inverted pendulum with one link from the ankle to the COM. We modeled the posture control system using feedback control with up to third-order nonlinear control (Fig. 4). Many studies of the cerebellum have indicated that the cerebellum generates feedforward control using an internal model^55^. Modeling studies for quiet standing have also indicated the contribution of open-loop (feedforward) control by using, for example, stabilogram diffusion analysis (SDA)^56,57^. However, simulation studies of the posture control model showed that simple feedback control can reproduce most characteristics of human standing, including SDA^16,54^. This indicates that the characteristics of the open-loop control do not explicitly participate in the behavior of the quiet standing of healthy humans and, thus, that most of the current studies of the posture control model use feedback-based control^58,59^. Previous studies of posture control with PID feedback have numerically evaluated the physiological characteristics of posture control, such as sensory reweighing^16,18^. Nonlinear control has been found to be necessary to explain the amplitude of body sway^21^. Large body sway is considered to occur due to cyclic motion^60^, and a nonlinear factor due to intermittent control is proposed to explain the underlying mechanism^22,58^. Third-order nonlinearity is the smallest-order nonlinearity that can produce the cyclic motion for large body sway^24^. To retain continuity and comparability with these previous results, we used feedback control with third-order nonlinearity. To confirm the ability of the model to explain the different properties between intact and IO rats, we analyzed the analytical solution of the model before it was used for identification.

We determined an analytical solution for the system with approximately zero noise. The system showed a periodic solution with angular frequencies *ω* and amplitudes *r* as Eqs. (1 and 2)*.* The COM of the inverted pendulum with the amplitude of the angle *r* sways with $h\sin r$ horizontally and, by assuming the movement to be constant, the SD becomes $1/\sqrt 2 { }h\sin r$.

The control parameters of the mathematical model were then identified. To do so, we performed a computer simulation of the model and searched for control parameters that fit the behavior of the simulation to that of the experiment. The time series of the elevation angle of the COM *θ* was obtained by the simulation and converted to the position of the COM using the trigonometric function *h* sin*θ*. Then, the PDF and PSD of the simulation were calculated from the obtained COM motion, using the same method as in the rat experiments. The control gains *k*_*P0*_, *k*_*P2*_, *k*_*D*_, and *k*_*I*_ and noise σ of the simulated model were taken as unknown parameters, and the simulation was repeatedly performed while these values were changed. Here, the delay time *Δ* was set at 0.04 s based on previous research^25^. The values of the unknown parameters were determined to minimize the difference between the PSD and PDF of the simulation and experiment. The Generic Algorithm was used for the parameter search.

### Ethical considerations

This study was approved by the Ethics Committee for Animal Experiments at the University of Tokyo and conducted in compliance with the Guidelines for Research with Experimental Animals of the University of Tokyo and the ARRIVE guidelines.

### Statistical analysis

To use rats as a unit and to avoid the multiple comparisons problem, we selected the data of one trial for each rat and used these values for the statistical analysis. The data selected for each rat were the most typical results of the intact/IO rats. To identify this “typical” trial, we calculated the PSD of all of the trials and the peak frequencies of the PSD. Then, we compared the peak frequencies for each trial with the average peak frequencies for intact/IO rats. We selected the trials with the closest peak frequencies to the average as the typical trial for intact/IO rats.

We evaluated statistical significance with a two-tailed test. The effects of 3-AP on the numbers of surviving neurons were tested using a two-way ANOVA of rat type (Intact/IOtest) and IO subdivision. The significances were also tested for each IO subdivision using a *t*-test between intact and IOtest rats. Coordination of body segments was calculated using the SVD, and differences in intersegmental coordination were assessed with two-way ANOVA of segment and rat type. The PDF of the COM was evaluated from its SD and kurtosis. The SD and kurtosis of intact and IO rats were compared using a *t*-test and Wilcoxon rank-sum test. The peak properties of the PSD of intact and IO rats were compared by testing the peak frequencies and amplitude in the peak frequencies using a *t*-test.

The identification of the control parameters of the model was repeated ten times and ten parameter sets were obtained for each rat. The convergence of identification was tested by comparing the variation in the ten parameter sets of the identification trials with the variation among different rats. Here, two-way ANOVA was applied to the identification trials and rats. After selection of the optimal parameter set from the ten parameter sets, two-way ANOVA with rat type and rats was used to test the significance of the difference between the parameters of the intact rats and those of the IO rats. Individual parameters were also compared between intact and IO rats using a *t*-test.

## Supplementary Information

Supplementary Information 1.

Supplementary Video 1.

## Data Availability

The measured data of standing rats, including body segment angles measured by the motion capture system, COM calculated from the motion capture data, and COP measured by the force plate, are available from the Dryad database (https://doi.org/10.5061/dryad.jwstqjq7x).
