# Supplementary material for: Quantitative evaluation of posture control in rats with inferior olive lesions
Source: Sci Rep. 2021 Oct 13;11:20362. doi: 10.1038/s41598-021-99785-w (PMC8514513; doi:10.1038/s41598-021-99785-w)
Supplement: Supplementary file 1 — Supplementary Information 1. [file 41598_2021_99785_MOESM1_ESM.pdf]

Supplementary Text

The magnitude of the body sway during standing was assessed using the standard deviation (SD) of the horizontal center of mass (COM) motion on the sagittal plane SD_{COM} in the main text. Table S1 shows the SD_{COM} for each intact rat and each IO rat. The same table also shows the SD of the center of pressure (COP) on the sagittal plane SD_{COP}. The mean (±SD) of the SD_{COM} was 1.54 (0.59) (mm) in the intact rats and 1.84 (0.78) (mm) in the IO rats. The mean (±SD) of the SD_{COP} was 2.74 (±0.24) (mm) in the intact rats and 2.97 (±0.68) (mm) in the IO rats. The SD_{COP} was significantly larger than the SD_{COM} (intact: $P < 0.001$, $t = -7.36$, $df = 10$; IO: $P = 0.002$, $t = -4.42$, $df = 9$, t-test). A previous study of the standing rat[1] assessed the magnitude of body sway using the COP, and the SD_{COP} was close to that reported in this article. Here, we discuss what factors contribute to the difference between the SD_{COM} and SD_{COP}.

By comparing the time series of the horizontal COM and COP on the sagittal plane (Fig. S1), we found that the COP showed faster oscillations than the COM. We then applied a low-pass filter (3 Hz, second-order Butterworth filter) to both the COM and COP and calculated the SD again (Table S1, COM < 3 Hz and COP < 3 Hz). The results showed that the SD of the COP decreased drastically, whereas the SD of the COM remained almost the same. There was no significant difference between the low-pass-filtered COM and COP (intact: $P < 0.76$, $t = 0.31$, $df = 10$; IO: $P < 0.63$, $t = -0.49$, $df = 9$, t-test). This result indicates the presence of a high-frequency component in the COP. This component is considered to underlie the difference between the COM and the COP.

Next, we considered what features of the motion generated the high-frequency component of the COP. When we model the standing motion with the inverted pendulum model shown in equation (S3), the following relationship between the COM and COP is supposed to exist for the balance around the ankle[2].

$$\mathrm{COM} - \mathrm{COP} = \frac{J}{mg}\ddot{\theta} \tag{S1}$$

This equation shows that the angular acceleration of the COM explains the difference between the COM and COP. Thus, we can calculate the COP from the COM and the angular acceleration of the COM obtained in the experiment by using the following equation.

$$\mathrm{COP}_{\mathrm{reconst}} = \mathrm{COM} - \frac{J}{mg}\ddot{\theta} \tag{S2}$$

The SDs of the reconstructed COP from COM: $COP_{reconst}$ are shown in Table S1. The obtained SD of the $COP_{reconst}$ was increased compared with the SD of the COM and they were close to the values of the SD of the COP. We tested the difference between the SD of the $COP_{reconst}$ and the SD of the COP and found no significant difference (intact: $P < 0.29$, $t = 1.11$, $df = 10$; IO: $P < 0.50$, $t = -0.71$, $df = 9$, t-test). These results suggest that the angular acceleration of the COM acts as a high-frequency component in the COP, which causes the difference between the COM and COP.

Supplementary Methods

Brain sections of rats After all of the experiments, the rats were deeply anesthetized, their chest opened, and physiological saline and 4% paraformaldehyde in 0.1 M phosphate buffer saline (PBS) was perfused via the heart to fix the brain. The extracted brain tissues were post-fixed as follows: the tissues were immersed in 4% paraformaldehyde in 0.1 M PBS for 24 hours, then immersed in 10% paraformaldehyde in 0.1 M PBS for 1 week, and then sequentially immersed in 5%, 10%, and 20% sucrose in 0.1 M PBS for 1 week each. The region containing the IONs was sectioned from the immersed brain tissue and 30-µm-thick coronal slices were prepared. The brain sections were stained with Nissl stain.

Experimental environment The experimental environment was constructed in our previous research[1]. To encourage the rats to keep standing, the water supply was placed at a height that required the rats to stand bipedally, and water was continuously provided to reward rats for standing. The force between the water supply and mouth of the rat was monitored with a three-dimensional force sensor (Tec Gihan, Japan). The tail of each rat was tethered to the environment with a string and connected with a load cell (Minebea, Japan) to prevent its use to support the bipedal posture and to monitor the force generated at the tail. A previous study of intact rats showed that, at maximum, about 50% and 10% of the torque required for stabilization was generated from the mouth and tail, respectively[1]. A force plate (TF-2020-A; Tec Gihan) was placed on the floor of the standing rat and the floor reaction force was measured at a measurement frequency of 1000 Hz.

Measurement of the motion A motion capture system (Oqus300+; Qualisys, Sweden) was placed around the rat to measure its movement. Reflective markers were attached to the rat's skin overlying 21 body landmarks. The markers at the center of the body were as follows: the top of the head (H1), the centers of the right and left scapulae (ShM), one- and two-thirds of the H1 and ShM (H2 and H3), and the center of the right and left iliac crests (HipM). Furthermore, the following markers were placed on the right and left sides of the body: scapulae (ShR and ShL), elbow joint (ElR and ElL), distal head of the ulna (ArmR and ArmL), iliac crest (HipR and HipL), greater trochanter (GtR and GtL), lateral condyle of the knee (KneeR and KneeL), lateral malleolus (AnkleR and AnkleL), and the distal end of the fifth metatarsal (MtR and MtL). The measurement frequency of the motion capture system was 500 Hz.

Analysis of intersegmental coordination Intersegmental coordination was analyzed using similar methods to those of previous studies on standing intact rats[1] and standing humans[3]. The time series of the elevation angles of the four body segments (trunk, thigh, shank, and foot) on the sagittal plane were calculated from the measured positions of the head, greater trochanter, knee, heel, and metatarsus. A matrix of the segmental motion was composed by arranging the time series of the elevation angles, and singular value decomposition (SVD) was applied to this matrix to derive the principle components of the segmental motion[1]. Through this decomposition, we can obtain the combination of body segments that are simultaneously active. This is called intersegmental coordination. To investigate the effect of an ION lesion on intersegmental

coordination, the component of the intersegmental coordination and the contribution rate of each intersegmental coordination to the movement were compared between intact and IO rats.

Analysis of the center of mass motion In our previous study, we measured the ratio between the COM of each body segment to the endpoints of the segment and the weight ratio of each segment to body weight in rats[1]. These data were used to calculate the position of the COM during standing. The endpoints of the trunk, thigh, shank, and foot were derived from the motion capture data. Here, the positions of the left and right body markers were averaged. Then, using the relationship between the endpoint position and the COM, the position of the COM of each body segment was obtained. The COM of the body was calculated by the weighted sum of the COM in each body segment.

The probability density function (PDF) of the COM position in the sagittal plane was derived from the measured COM motion and compared between intact and IO rats. The PDF of COM assesses the features of the distribution of the COM similarly to the histogram of the COM. In contrast to the discrete bin-by-bin existence probability obtained by histogram, the PDF derived using kernel distribution can represent a continuous distribution of the COM. We used the "ksdensity" function in MATLAB to compute the PDF.

To show the frequency characteristics of body sway, we derived the power spectrum density (PSD) of the COM motion. The PSD was calculated using the maximum entropy method (MEM), which is stable in the estimation in the low frequency range (see Funato *et al.* 2017[1] for a comparison of MEM and fast Fourier transform for PSD estimation in rats). To calculate the MEM, the Burg method with 512 dimensions was used[4, 5]. Because the COM movement in rats during bipedal standing had a characteristic frequency around 1 Hz[1], the PSD was analyzed in the range of 0.05–10 Hz.

Mathematical model of posture control in rats We model the body motion of the standing rat using an inverted pendulum with one link from the ankle to the COM as follows.

$$J\ddot{\theta} = mgh\theta + \tau \tag{S3}$$

where θ is the elevation angle of the body from the vertical, m, h, J, and g are the mass, length, and moment of inertia of the pendulum and acceleration due to gravity, respectively, and τ is the control torque to maintain posture. We assume the following feedback control model with up to third-order nonlinear control terms (Fig. 4).

$$\begin{cases} \tau \ = -k_P\theta_\Delta - k_D\dot{\theta}_\Delta - k_I\int \theta_\Delta dt + \sigma\xi \\ k_P = k_{P0} + k_{P1}\theta_\Delta + k_{P2}\theta_\Delta^2 \end{cases} \tag{S4}$$

where θ_Δ is θ containing the sensory delay, k_P, k_D, and k_I are the proportional, differential, and integral control gain, respectively, and $\sigma\xi$ is the noise. The second-order nonlinear control term k_{P1} acts on the skewness of the COM distribution (the difference between forward and backward motion). Here, the skewness in the rat COM distribution seemed minor (Fig. 3A) and, to keep the model simple, we set $k_{P1} = 0$.

Analysis of the mathematical model From the equations of motion (Eq. (S3)) and of the posture control model (Eq. (S4)), the equation for the standing motion of rat is represented by the following equation.

$$J\ddot{\theta} = mgh\theta - \left(k_{P0} + k_{P1}\theta_\Delta + k_{P2}{\theta_\Delta}^2\right)\theta_\Delta - k_D\dot{\theta}_\Delta - k_I\int \theta_\Delta dt + \sigma\xi \tag{S5}$$

Here, as described in the main text, we set k_{PI} = 0 and the noise small (σ=0). Accordingly, the equation becomes

$$J\ddot{\theta} = mgh\theta - \left(k_{P0} + k_{P2}{\theta_\Delta}^2\right)\theta_\Delta - k_D\dot{\theta}_\Delta - k_I\int \theta_\Delta dt \tag{S6}$$

From Eq. (S6), we consider the cyclic solution that generates body sway. To focus on the solution by conjugate poles in Eq. (S6), we use the following general form of the solution.

$$\begin{cases} \text{solution:} & \theta = ae^{j\omega t} + a^* e^{-j\omega t} \\ \text{restraint condition:} & \dot{a}e^{j\omega t} + \dot{a}^* e^{-j\omega t} = 0 \end{cases} \tag{S7}$$

Here, for the purpose of simplicity, the integral term is approximated by

$$\int \theta dt = \frac{1}{j\omega}\left(ae^{j\omega t} - a^* e^{-j\omega t}\right) = 0. \tag{S8}$$

Substituting Eqs. (S7) and (S8) into Eq. (S6), the equation becomes

$$2jJ\omega\dot{a}e^{j\omega t} - J\omega^2\theta = mgh\theta - k_{P0}\theta_\Delta - k_{P2}{\theta_\Delta}^3 - k_D j\omega\theta_\Delta^* - \frac{k_I}{j\omega}\theta_\Delta^* \tag{S9}$$

and by multiplying $e^{-j\omega t}$ by both members, the above equation becomes

$$2jJ\omega\dot{a} = (mgh + J\omega^2)\theta e^{-j\omega t} - k_{P0}\theta_\Delta e^{-j\omega t} - k_{P2}{\theta_\Delta}^3 e^{-j\omega t} - \frac{k_I - \omega^2 k_D}{j\omega}\theta_\Delta^* e^{-j\omega t} \tag{S10}$$

Here, from the following relationship

$$\begin{cases} \theta_\Delta e^{-j\omega t} = ae^{-j\omega\Delta} + a^* e^{-2j\omega t}e^{j\omega\Delta} \\ \theta_\Delta^* e^{-j\omega t} = ae^{-j\omega\Delta} - a^* e^{-2j\omega t}e^{j\omega\Delta} \\ \theta_\Delta^3 e^{-j\omega t} = a^3 e^{2j\omega t}e^{-3j\omega\Delta} + 3a^2 a^* e^{-j\omega\Delta} + 3aa^{*2}e^{-2j\omega t}e^{j\omega\Delta} + a^{*3}e^{-4j\omega t}e^{3j\omega\Delta} \end{cases} \tag{S11}$$

Eq. (S10) becomes

$$\begin{aligned} 2jJ\omega\dot{a} = {} & (mgh + J\omega^2)\left(a + a^* e^{-2j\omega t}\right) - k_{P0}\left(ae^{-j\omega\Delta} + a^* e^{-2j\omega t}e^{j\omega\Delta}\right) \\ & - \frac{k_I - \omega^2 k_D}{j\omega}\left(ae^{-j\omega\Delta} - a^* e^{-2j\omega t}e^{j\omega\Delta}\right) \\ & - k_{P2}\left(a^3 e^{2j\omega t}e^{-3j\omega\Delta} + 3a^2 a^* e^{-j\omega\Delta} + 3aa^{*2}e^{-2j\omega t}e^{j\omega\Delta} + a^{*3}e^{-4j\omega t}e^{3j\omega\Delta}\right) \end{aligned} \tag{S12}$$

By integrating Eq. (S12) for one cycle, the equation becomes

$$\dot{a} = -\frac{aj}{2J\omega}(mgh + j\omega^2) + \frac{aj}{2J\omega}(k_{P0} + 3k_{P2}aa^*)e^{-j\omega\Delta} + \frac{k_I - \omega^2 k_D}{2J\omega^2}ae^{-j\omega\Delta} \tag{S13}$$

Using the relationship $e^{-j\omega\Delta} = \cos\Delta\omega - j\sin\Delta\omega$, Eq. (S13) becomes

$$\dot{a} = \frac{a}{2J\omega}\left\{(k_{P0} + 3k_{P2}aa^*)\sin\Delta\omega + \frac{k_I - \omega^2 k_D}{\omega}\cos\Delta\omega\right\}$$

$$-\frac{aj}{2J\omega}\left\{J\omega^2 + mgh - (k_{P0} + 3k_{P2}aa^*)\cos\Delta\omega + \frac{k_I - \omega^2 k_D}{\omega}\sin\Delta\omega\right\} \qquad \text{(S14)}$$

We can extract a cyclic solution from this equation. From the cyclic solution $a = re^{j\varphi}$ and Eq. (S14),

$$\dot{a} = \dot{r}e^{j\phi} + j\dot{\phi}re^{j\phi}$$

$$= \frac{r}{2J\omega}\left\{(k_{P0} + 3k_{P2}r^2)\sin\Delta\omega + \frac{k_I - \omega^2 k_D}{\omega}\cos\Delta\omega\right\}e^{j\phi}$$

$$-\frac{jr}{2J\omega}\left\{J\omega^2 + mgh - (k_{P0} + 3k_{P2}r^2)\cos\Delta\omega + \frac{k_I - \omega^2 k_D}{\omega}\sin\Delta\omega\right\}e^{j\phi} \qquad \text{(S15)}$$

By comparing the real and imaginary parts of Eq. (S15), the following relationship is obtained.

$$\begin{cases} \dot{r} = \frac{r}{2J\omega}\left\{(k_{P0} + 3k_{P2}r^2)\sin\Delta\omega + \frac{k_I - \omega^2 k_D}{\omega}\cos\Delta\omega\right\} \\ \dot{\phi} = -\frac{1}{2J\omega}\left\{J\omega^2 + mgh - (k_{P0} + 3k_{P2}r^2)\cos\Delta\omega + \frac{k_I - \omega^2 k_D}{\omega}\sin\Delta\omega\right\} \end{cases} \qquad \text{(S16)}$$

Here, we consider that $\Delta\omega$ is small, namely, $\sin\Delta\omega \cong \Delta\omega$ and $\cos\Delta\omega \cong 1$, and the equation becomes

$$\begin{cases} \dot{r} = \frac{r}{2J\omega^2}(k_{P0}\Delta\omega^2 + 3k_{P2}r^2\Delta\omega^2 + k_I - \omega^2 k_D) \\ \dot{\phi} = -\frac{1}{2J\omega}(J\omega^2 + mgh - k_{P0} - 3k_{P2}r^2 + \Delta k_I - \Delta\omega^2 k_D) \end{cases}$$

From the above equations with $\dot{r} = 0$, $\dot{\phi} = 0$, the following cyclic solution is obtained.

$$\begin{cases} \omega^2 = \frac{k_D - k_I\Delta^2 - mgh\Delta \pm \sqrt{(k_D - k_I\Delta^2 - mgh\Delta)^2 - 4\Delta k_I(J - \Delta k_D)}}{2\Delta(J - \Delta k_D)} \\ r^2 = \frac{-2k_{P0}\Delta + k_D + k_I\Delta^2 + mgh\Delta \pm \sqrt{(k_D - k_I\Delta^2 - mgh\Delta)^2 - 4\Delta k_I(J - \Delta k_D)}}{6k_{P2}\Delta} \end{cases} \qquad \text{(S17)}$$

The equation of angular frequency ω shows that the frequency of the periodic solution is determined by the differential control gain k_D and the integral control gain k_I. Furthermore, the integral control gain k_I has a relatively smaller effect than k_D because the square of the $\Delta \cong 0.04$ s [6] works with k_I. Therefore, approximating $k_I \cong 0$, we obtain

$$\omega^2 \simeq \frac{k_D - mgh\Delta}{\Delta(J - \Delta k_D)}. \qquad \text{(S18)}$$

The same approximation of $k_I \cong 0$ for the amplitude r yields

$$r^2 \simeq \frac{k_D - k_{P0}\Delta}{3k_{P2}\Delta}. \qquad \text{(S19)}$$

Identification of control parameters To investigate the behavior of the mathematical model, we performed a computer simulation. A numerical calculation of the equation of motion including noise (stochastic differential equation) was performed by using the Euler-Maruyama method. The sampling frequency was 200 Hz and the duration of the simulation was 180 s. The time series of the elevation angle of the COM θ

was obtained by the simulation and it was converted to the position of the COM using the trigonometric function h sinθ. Then, the PDF and PSD of the simulation were calculated from the COM motion obtained, applying the same method used for the rat experiments.

The quantitative evaluation of the control system, that is, identification, was performed by a comparison of the behavior in the simulation and rat experiments. The control gains k_{P0}, k_{P2}, k_D, and k_I and noise σ of the simulated model were taken as unknown parameters, and we repeatedly performed the simulation while changing these values. Here, the delay time Δ was set as 0.04 s based on previous research[6]. The values for the unknown parameters were determined so that the PSD and PDF of the simulation and experiment were as close as possible. The evaluation function was set as the sum of the root mean square (RMS) of the difference in the PDF and RMS of the logarithmic difference in the PSD. We searched for parameters that minimized the evaluation function. The Generic Algorithm was used for the parameter search. We used the "ga" function in MATLAB. To assess the convergence of the parameters, the identification was repeated ten times with different random seeds, and ten types of parameters were obtained for each experimental trial. Next, we tested the convergence of the identification by comparing the variation in the ten parameter sets obtained with the variation among experimental trials. Then, the optimal parameter set with the smallest evaluation function among the ten identification trials was adopted as the parameters of the model. The optimal parameter sets for intact and IO rats were compared to consider the effect of the ION lesion.

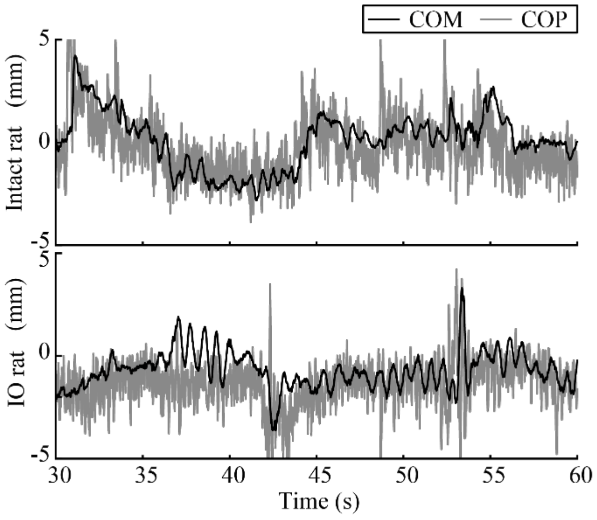

Fig. S1 Comparison of the COM and COP in horizontal motion during standing. Each chart represents one trial of an intact rat and that of an IO rat. For ease of visualization, a low-pass filter (30 Hz, second-order Butterworth filter) was applied to the COP data.

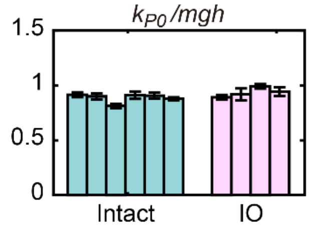

	Rat	ID	Rat	ID
SD	0.039	0.024	0.49	0.02

	Rat	ID	Rat	ID
P	<0.001	0.40	<0.001	0.05
F	24.6	1.07	17.4	2.28
df	5	9	3	9

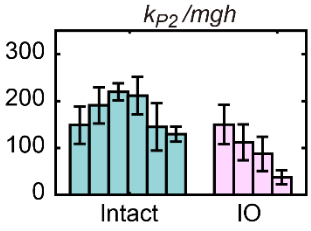

	Rat	ID	Rat	ID
SD	38.2	34.1	62.2	22.0

	Rat	ID	Rat	ID
P	<0.001	0.71	<0.001	0.05
F	10.49	0.70	24.5	2.28
df	5	9	3	9

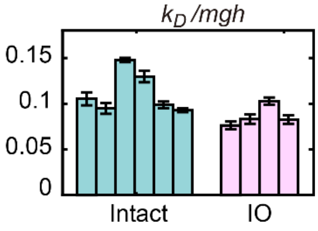

	Rat	ID	Rat	ID
SD	0.022	0.005	0.046	0.003

	Rat	ID	Rat	ID
P	<0.001	0.06	<0.001	0.05
F	237.4	2.05	83.5	2.26
df	5	9	3	9

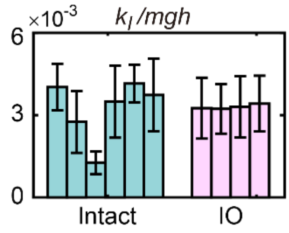

	Rat	ID	Rat	ID
SD	1.0×10^{-3}	1.0×10^{-3}	1.7×10^{-3}	0.7×10^{-3}

	Rat	ID	Rat	ID
P	<0.001	0.82	0.98	0.85
F	10.7	0.57	0.06	0.52
df	5	9	3	9

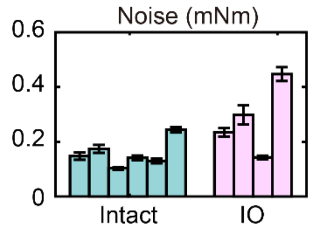

	Rat	ID	Rat	ID
SD	0.31	0.07	1.15	0.09

	Rat	ID	Rat	ID
P	<0.001	0.22	<0.001	0.13
F	185.0	1.38	309.0	1.75
df	5	9	3	9

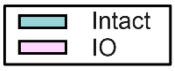

Fig. S2 Identified control parameters. The figures show the result of linear control gain k_{P0}, nonlinear control gain k_{P2}, differential control gain k_D, integral control gain k_I, and noise magnitude. Parameter values were normalized by *mgh* (*m* mass, *g* acceleration due to gravity, and *h* body height). Each bar shows the average and standard deviation of identification trials (n = 10). The tables below the bar charts are the standard deviation (SD) and the result of two-way ANOVA with rat (Rat) and identification trial (ID). The standard deviation of the identification trials are the mean SD of each rat. The ANOVA results are described with P values (P), F values (F), and degrees of freedom (df). P values exceeding 0.05 are written with a green background.

Table S1 Standard deviation (SD) of the time series in the COM and COP. COM < 3 Hz and COP < 3 Hz represent the SD of the COM and COP after low-pass filtering with a 3-Hz threshold. $COP_{reconst}$ represents the SD of the COP estimated from the COM. Data are those of six intact rats (11 trials) and four IO rats (10 trials). Mean (SD) shows the mean and SD of the 11 trials in the intact rats and the ten trials in the IO rats.

	Intact Rat								
	R1		R2	R3		R4			
COM	1.92	1.11	1.31	0.75	1.49	1.50	0.91	2.01	1.07
COP	2.49	2.98	2.74	2.73	2.72	2.52	2.56	3.16	2.43
COM < 3 Hz	1.89	1.07	1.26	0.79	1.62	1.45	0.91	1.99	1.05
COP < 3 Hz	1.47	1.94	1.48	1.09	1.51	0.84	1.38	2.27	0.99
$COP_{reconst}$	2.58	1.83	1.77	0.99	2.67	2.06	1.57	2.55	2.48

	Intact Rat		
	R5	R6	Mean (SD)
COM	2.41	2.46	1.54 (0.59)
COP	2.72	3.05	2.74 (0.24)
COM < 3 Hz	2.38	2.34	1.52 (0.56)
COP < 3 Hz	1.21	1.86	1.46 (0.43)
$COP_{reconst}$	5.04	4.28	2.53 (1.18)

	IO Rat										
	IO1			IO2		IO3		IO4			Mean (SD)
COM	1.43	1.84	1.28	1.89	1.56	1.10	0.92	3.34	2.96	2.05	1.84 (0.78)
COP	2.14	2.25	2.40	4.38	3.26	2.89	2.86	3.70	3.01	2.78	2.97 (0.68)
COM<3Hz	1.24	1.75	1.17	1.74	1.47	1.11	0.91	3.24	2.59	2.03	1.72 (0.73)
COP<3Hz	1.37	1.65	1.78	3.12	1.79	1.75	1.33	2.56	1.72	1.41	1.85 (0.57)
COP Reconst	1.98	2.27	1.89	2.54	6.16	1.54	1.72	6.00	6.35	4.06	3.45 (2.00)

Table S2 Body mass and height of the rats used in the behavioral experiments. Body height was measured from the floor to the head of the rats. Because wild 1 and IO1 were the same rat, nine rats were used in total in the behavioral experiments.

	Intact rats					
	Wild 1	Wild 2	Wild 3	Wild 4	Wild 5	Wild 6
Mass (g)	222.6	201.4	198.2	218.3	173.9	190.2
Height (mm)	173.6	125.3	154.8	159.5	162.0	173.8

	IO rats			
	IO1	IO2	IO3	IO4
Mass (g)	305.4	188.4	216.4	206.5
Height (mm)	177.8	158.7	154.4	176.9

Video S1

The movie shows the standing motion of intact and IO rats. Rats stood on only their hindlimbs, and their behaviors were measured using a motion capture system.

SI References

1. Funato, T. *et al.* Postural control during quiet bipedal standing in rats. *PLoS One.* **12**, e0189248 (2017).
2. Jacono, M., Casadio, M., Morasso, P.G. & Sanguineti, V. The sway-density curve and the underlying postural stabilization process. *Motor Control.* **8**, 292-311 (2004).
3. Pinter, I.J., van Swigchem, R., van Soest, A.J. & Rozendaal, L.A. The dynamics of postural sway cannot be captured using a one-segment inverted pendulum model: a PCA on segment rotations during unperturbed stance. *J Neurophysiol.* **100**, 3197-3208 (2008).
4. Sawada, Y. *et al.* New technique for time series analysis combining the maximum entropy method and non-linear least squares method: its value in heart rate variability analysis. *Med Biol Eng Comput.* **35**, 318-322 (1997).
5. Aoki, M. *et al.* A characteristic pattern in the postural sway of unilateral vestibular impaired patients. *Gait Posture.* **40**, 435-440 (2014).
6. Muramatsu, H. *et al.* Evoked potentials elicited on the cerebellar cortex by electrical stimulation of the rat spinocerebellar tract. *Surg Neurol.* **72**, 395-400 (2009).
